# CLRM-03. BGB-290 AND TEMOZOLOMIDE IN TREATING ISOCITRATE DEHYDROGENASE (IDH)1/2-MUTANT GRADE I-IV GLIOMAS – A NOVEL MODEL OF AYA TRIAL DEVELOPMENT AND DEPLOYMENT

**DOI:** 10.1093/noajnl/vdab112.002

**Published:** 2021-09-21

**Authors:** Asher Marks, Ranjit Bindra

**Affiliations:** Yale University, New Haven, CT, USA

## Abstract

**DESCRIPTION:**

The lack of enrollment of AYA patients on clinical trials is well documented and multivariant. Here we present the basic science, examination of its relevance to the AYA population specifically, and the parallel deployment of two international clinical trials via a pediatric neuro-oncology and adult brain tumor consortium.

**DISCUSSION:**

In February of 2017, the laboratory of Ranjit Bindra, MD, PhD, published a manuscript describing the finding that tumors with IDH1/2 mutations induce a BRCAness state leading to PARP inhibitor (PARPi) sensitivity and synergistic interactions with temozolomide chemotherapy [2]. Despite IDH1/2 mutations being rare in the pediatric high-grade glioma population, three independent groups confirmed that the incidence is significantly increased to ~30% in the adolescent and young adult (AYA) population. Upon discovery of a high blood-brain-barrier penetrant, high potency PARPi by BeiGene Pharmaceuticals, an international trial was launched through the Pacific Pediatric Neuro-Oncology Consortium (PNOC) [3] to test this drug in an AYA specific trial recruiting patients ages 13 to 25, with a concurrent trial being run for patients older than 25 years of age through the Adult Brain Tumor Consortium (ABTC) [4].

While most trials that enroll AYA patients are forced to assess them as a unique cohort in post-analysis, if at all, the PNOC trial mentioned above was designed from the ground up with the AYA population in mind. It has allowed us to base initial dosing, recruitment strategies, psychosocial assessments, and outcomes, specifically on the AYA population. Ultimately, we expect their distinctive biology to yield unique results when compared to the ABTC trial.

We propose that this is a model that could potentially be replicated in other disease processes and early phase drugs with the buy-in of the pharmaceutical industry and early phase consortiums.

Acknowledgements: BeiGene Pharmaceuticals, PNOC, ABTC, CureSearch, Gateway Foundation

